# Genetic parameters for somatic cell score, milk yield and type traits in Nigerian Dwarf goats

**DOI:** 10.5713/ab.21.0143

**Published:** 2021-06-23

**Authors:** Mauricio Valencia-Posadas, Alma Arianna Lechuga-Arana, Fidel Ávila-Ramos, Lisa Shepard, Hugo H. Montaldo

**Affiliations:** 1 Departamento de Veterinaria y Zootecnia, División de Ciencias de la Vida, Campus Irapuato-Salamanca, Universidad de Guanajuato, Irapuato, Guanajuato, CP 36824, México; 2 Programa de Doctorado en Biociencias, División de Ciencias de la Vida, Campus Irapuato-Salamanca, Universidad de Guanajuato, Irapuato, Guanajuato, CP 36824, México; 3 American Dairy Goat Association, Spindale, NC 28160, USA; 4 Departamento de Genética y Bioestadística, Facultad de Medicina Veterinaria y Zootecnia, Universidad Nacional Autónoma de México, Ciudad Universitaria, Ciudad de México, 04510, México

**Keywords:** Fat Yield, Genetic Correlation, Goats, Heritability, Protein Yield

## Abstract

**Objective:**

This study was conducted to estimate multi-trait genetic parameters for somatic cell score (SCS), milk yield and type traits in Nigerian Dwarf (ND) goats from the United States.

**Methods:**

Data from 1,041 ND goats in the United States with kiddings in 95 herds were used to estimate multi-trait genetic parameters for SCS, milk (MILK), fat (FAT), and protein (PROT) yields, and 14 type traits. An 18-trait mixed linear animal model for lactation mean SCS (Log_2_), MILK, FAT, PROT, and 14 type traits was applied. A factor analytic approach (FA1) in ASReml software was used to obtain convergence.

**Results:**

Averages for SCS were low (2.85±1.29 Log_2_), and were 314±110.6, 20.9±7.4, and 14±4.9 kg, respectively, for MILK, FAT, and PROT. Heritabilities for SCS, MILK, FAT, and PROT were 0.32, 0.16, 0.16, and 0.10, respectively. The highest heritabilities for type traits were for stature (0.72), teat diameter (0.49), and rump width (0.48), and the lowest estimates were for dairyness (0.003) and medial suspensory ligament (0.03). Genetic correlations of SCS with MILK, FAT, and PROT were positive but low (0.25, 0.18, and 0.23, respectively). Genetic and phenotypic correlations between MILK, FAT, and PROT were high and positive (≥0.66). Absolute values of genetic correlations involving SCS with type traits were generally low or no different from zero. Most of the phenotypic correlations involving SCS with type traits were low. No serious unfavorable genetic correlations between milk yield traits and SCS or between milk yield traits or SCS and type traits were found.

**Conclusion:**

Genetic variation exists in the ND breed for most studied traits. The development of selection programs based on these estimates may help accelerate favorable multi-trait genetic changes in this breed.

## INTRODUCTION

Reliable estimates of genetic parameters, including heritability and genetic (co)variances, are crucial to the establishment of efficient genetic improvement programs and development of multi-trait selection indices [1]. Multi-trait analyses can consider the correlation structure among all traits and this in turn increases the accuracy of evaluation and reduces selection bias. Therefore, their use could be an effective strategy to obtain reliable and unbiased parameter estimates [2]. The Nigerian Dwarf (ND) breed is a small goat originally from Africa now used in the United States (US). It is derived from imports of landrace-type goats from Nigeria in the 1950s and 1960s. The American Dairy Goat Association (ADGA) herdbook was established in 1987 but the ND was not included until 2005. There is also an American Nigerian Dwarf Dairy Association that does not register animals but supports registration with the ADGA. Dairy goat production in the United States is comparatively small but well established and stable in numbers [3]. Most ND goats are for home-scale dairy production. Due to economies of scale, there are few commercial dairies solely dedicated to this breed, although some operations use it as a supplement to other breeds to increase components for a higher cheese yield. Some goat breeders in coordination with ADGA technicians routinely record milk traits, somatic cell count (SCC) and evaluate the goats for type [3].

Few joint multi-trait quantitative genetic analyses of somatic cell score (SCS), milk and type traits have been conducted simultaneously in goats [4]. Data on heritabilities, genetic and phenotypic correlations for SCS, milk, fat and protein yield and type traits in ND goats using multi-trait models in the US or worldwide is practically absent. Moreover, the ND breed is different from other breeds in the US due to its distinctly smaller size. Evidence of a high genetic distance has been found between the West African Dwarf, considered the predecessor population of ND, and Saanen goats based on DNA marker analysis [5]. Research to propitiate genetic improvement programs in exotic, less common breeds that are genetically distant from commercial lines is fundamental in order to increase or maintain genetic diversity [6]. The objective of our study was to estimate multi-trait genetic parameters for SCS, milk yield and type traits in ND goats from the United States.

## MATERIALS AND METHODS

### Data and variables

In this analysis, we used data from 1,041 ND goats registered by the ADGA with kiddings in 95 US herds from 2005 to 2015. Animals were evaluated for the following 14 type traits: final score (FIS), stature (STA), strength (STR), dairyness (DAI), rump angle (RUA), rump width (RUW), rear legs (REL), fore udder attachment (FUA), rear udder height (RUH), rear udder arch (RUC), medial suspensory ligament (SUS), udder depth (UDD), teat placement (TEP), and teat diameter (TED), using the ADGA appraisal system. A scale of 50 to 99 points was used for FIS, while a scale of 1 to 50 points was used for all other (linear) type traits [3,7].

Milk samples were collected as part of the official national milk recording system used by ADGA. Individual data on milk yield were obtained monthly during the complete lactation by using equipment built into the milking system according to Dairy Herd Improvement Association standards. Milk analysis included SCC using Soma Count laser-based flow-cytometry equipment calibrated with cow milk [3]. Fat and protein contents (F% and P%, respectively) were determined using Bentley infrared analyzer equipment. In order to have a variable close to a normal distribution, SCC was transformed to a linear scale from 0 to 9 based on a transformed variable defined as SCS. The formula used for the transformation was SCS = Log_2_ (SCC/100,000)+3 [3,8,9]. For SCS, each goat’s lactation record was obtained as the mean of monthly test-day records of up to 305 days. The average number of tests per record (standard deviation) was 6.7 (2.2). Lactation MILK, FAT, and PROT data were corrected to a 305-day mature equivalent basis by the USDA [8]. All type traits were previously corrected for age at appraisal [10].

### Data edition

Animals with incomplete information were eliminated. Only one record was retained for each animal in the final file, since the small numbers of animals with repeated records do not allow for an accurate estimate of a permanent environmental effect, which could have compromised the unbiasedness of the genetic parameter estimates. Records from animals with less than three test day records for milk traits and SCS and from herds with less than three records were also deleted.

Pedigree was verified and ordered and was pruned to keep only connected ancestors up to two generations back from animals with records. The final file contained a total of 2,762 ND animals. Excluding the base animals, 2.7% of sires and 4.9% of dams were unknown.

### Statistical analysis

The model used in the analysis was an 18-trait mixed linear animal model for mean SCS (Log_2_), MILK, FAT and PROT [8] and 14 type traits (FIS, STA, STR, DAI, RUA, RUW, REL, FUA, RUH, RUC, SUS, UDD, TEP, and TED).

In mathematical terms the model was defined as:


$$\begin{array}{c} \left[\begin{array}{c} y_{1}\\ \vdots \\ y_{18} \end{array}\right]=\left[\begin{array}{ccc} X_{1} & \ldots & 0\\ \vdots & \ddots & \vdots \\ 0 & \ldots & X_{18} \end{array}\right]\;\left[\begin{array}{l} b_{1}\\ \vdots \\ b_{18} \end{array}\right]+\left[\begin{array}{ccc} Z_{1} & \ldots & 0\\ \vdots & \ddots & \vdots \\ 0 & \ldots & Z_{18} \end{array}\right]\;\left[\begin{array}{l} a_{1}\\ \vdots \\ a_{4} \end{array}\right]\\ +\left[\begin{array}{ccc} H_{1} & \ldots & 0\\ \vdots & \ddots & \vdots \\ 0 & \ldots & H_{18} \end{array}\right]\;\left[\begin{array}{c} h_{1}\\ \vdots \\ h_{18} \end{array}\right]+\left[\begin{array}{l} e_{1}\\ \vdots \\ e_{18} \end{array}\right] \end{array}$$

Where: **y_1_**…**y_18_** are the vectors of observations for analysed traits. **X_1_**…**X_18_** are the incidence matrices that relate the fixed effects to observations; **b_1_**…**b_18_** are the vectors of fixed effects; **Z_1_**…**Z_18_** are the incidence matrices that relate the random animal additive genetic effects to observations; **a_1_**…**a_18_** are the vectors of random animal additive genetic effects; **H_1_**…**H_18_** are the incidence matrices that relate the random herd-year effects to observations; **h_1_**…**h_18_** are the vectors of herd-year effects; and **e_1_**…**e_18_** are the vectors of random residual effects.

Expectations (E) and (co)variance matrices (Var) of the model effects are defined in the following equations:


$$\begin{array}{l} \text{E}\;\left[\begin{array}{c} y\\ a\\ h\\ e \end{array}\right]=\left[\begin{array}{c} X\beta \\ 0\\ 0\\ 0 \end{array}\right];\text{Var}\;\left[\begin{array}{c} a_{1}\\ \vdots \\ a_{18} \end{array}\right]=A⊗\left[\begin{array}{ccc} \sigma_{{\text{a}}_{1}}^{2} & \ldots & \sigma_{{\text{a}}_{1,18}}\\ \vdots & \ddots & \vdots \\ \sigma_{{\text{a}}_{1,18}} & \ldots & \sigma_{{\text{a}}_{4}}^{2} \end{array}\right];\\ \text{Var}\;\left[\begin{array}{c} h_{1}\\ \vdots \\ h_{18} \end{array}\right]=\left[\begin{array}{ccc} \sigma_{{\text{h}}_{1}}^{2} & \ldots & \sigma_{{\text{h}}_{1,18}}\\ \vdots & \ddots & \vdots \\ \sigma_{{\text{h}}_{1,18}} & \ldots & \sigma_{{\text{h}}_{18}}^{2} \end{array}\right];\text{Var}\;\left[\begin{array}{c} e_{1}\\ \vdots \\ e_{18} \end{array}\right]=\left[\begin{array}{ccc} \sigma_{{\text{e}}_{1}}^{2} & \ldots & \sigma_{{\text{e}}_{1,18}}\\ \vdots & \ddots & \vdots \\ \sigma_{{\text{e}}_{1,18}} & \ldots & \sigma_{{\text{e}}_{18}}^{2} \end{array}\right]. \end{array}$$

Where: **A** is the numerator additive relationship matrix, ⊗ is the Kronecker product operator and $\sigma_{{\text{a}}_{\text{i}}}^{2},\;\sigma_{{\text{h}}_{\text{i}}}^{2},\;\sigma_{{\text{e}}_{\text{i}}}^{2},\;\sigma_{{\text{a}}_{\text{ij}}},\;\sigma_{{\text{h}}_{\text{ij}}}$, σ_a_ij__, σ_h_ij__ and σ_e_ij__ are scalars.

The model for SCS (Log_2_), MILK, FAT, and PROT included fixed effects of year of kidding, lactation number (1, 2, 3, and ≥4), kidding season (1 = January and February plus a few kiddings between June and December; 2 = March; and 3 = April and May). A Legendre regression polynomial of order 3 for average days in milk was applied as fixed effect for SCS only. The model for type traits included the fixed effects of the linear and quadratic effects of age at appraisal, plus the effect of month of appraisal which included five levels from May to September. Some data from April and October were classified together with the May and September levels, respectively. Random correlated herd-year, animal and error terms were fitted for all traits. The inverse of the numerator additive genetic relationship matrix **A** was used to estimate the variances and covariances in the model. Genetic parameters were obtained using the sum of animal and error variance as estimates of phenotypic variance similar to García-Peniche et al [11].

Analyses were performed with AI-Reml using ASReml v 4.1 [12]. A factor analytic approach (FA1) for animal and herd-year effects was used to obtain convergence for this high dimension problem [13]. At convergence, unstructured variance-covariance matrices were obtained from FA1 solutions with a final additional iteration. Approximate significance for genetic correlations different from 0 was obtained with one-tailed tests and assuming normality and α = 0.05 [14].

## RESULTS

The averages for SCS, and 305-day MILK, FAT, and PROT were 2.85±1.29 Log2, and 314±110.6 kg, 20.9±7.4 kg, 14±4.9 kg, respectively (Table 1). Average fat and protein content were 6.66% and 4.45%, respectively. The coefficient of variation for the three yield traits was moderate (around 35%). For type traits, the highest and lowest average scores obtained in this study were for FUA (31.3 points) and TED (11.28 points). This low score is probably due to the small size of ND goats compared to other breeds. The lowest coefficient of variation was found for FIS (3.88%) and the highest for TED (58.52%).

Heritabilities for SCS, MILK, FAT, and PROT were 0.32, 0.16, 0.16, and 0.10, respectively (Table 2). The SEs are relatively high due to sample size and perhaps data structure but are nonetheless probably acceptable as an initial point of reference. We chose a final model that included covariances between random herd-year effects across traits that yielded lower estimates for MILK, FAT, and PROT compared to models with independent herd-year effects. The idea was to obtain more robust estimates that are not inflated, which is a common problem with goat data from small herds [11]. The highest heritabilities for type traits were for STA (0.72), TED (0.49), and RUW (0.48), and the lowest estimates were for DAI (0.003) and SUS (0.03). Heritability estimates for milk yield traits were in a narrow range and were lower than that for SCS, while those for type traits were in a wide range, revealing the peculiarities of this population.

Genetic correlations between SCS with MILK, FAT, and PROT were all unfavorable (positive) with values between 0.18 and 0.25 (Table 3). The genetic and phenotypic correlations between MILK, FAT, and PROT were all high and positive (≥0.66). Absolute values of genetic correlations involving SCS and type traits were generally low with a maximum value of 0.22 for SUS, and high estimated standard errors (SEs). A wide range of genetic correlation values were observed between type traits with milk yield traits, from 0.88 between SUS with MILK, to 0.01 between FAT with REL and FAT with TEP. High and positive genetic correlations were found between SUS with MILK, FAT, and PROT (0.88, 0.64, and 0.81, respectively), between DAI with MILK, FAT, and PROT (0.70, 0.51, and 0.64, respectively) and between RUW with the same milk yield traits, with values of 0.76, 0.55, and 0.70, respectively. Genetic correlations between FIS with MILK, FAT, and PROT were −0.52, −0.38, and −0.48 respectively, and 0.72, 0.52, and 0.65, respectively, between STA with these same milk yield traits. Other slightly high negative genetic correlations were observed between RUH with MILK (−0.64), FAT (−0.47), and PROT (−0.59). We found moderate to low positive genetic correlations between STR, TED, and UDD with milk yield traits of 0.17 to 0.37, and negative correlations between RUA and FUA with the same milk yield traits (−0.12 to −0.24).

Moderate negative values of genetic correlations were found between FIS with SUS, RUW, STA, and DAI (−0.36 to −0.46), and a positive genetic correlation was observed with RUH (0.33) (Table 3). Positive and high genetic correlations were found between SUS with RUW, STA and DAI (0.67, 0.63, and 0.62, respectively), and between DAI with RUW and STA, whose values were 0.53 and 0.50, respectively, but negative with RUH (−0.45). Moderate to high values were found between RUW and STA (0.55) and RUW with RUH (−0.49), and low genetic correlations close to zero were observed between TEP with all traits, from −0.01 to 0.02.

High and positive phenotypic correlations were found between milk yield traits (≥0.84), with moderate to low correlations between FIS, STA, DAI, RUW, RUC, SUS, and FUA with milk yield traits (from 0.12 to 0.33) and negative correlations between UDD with milk yield traits (approximately −0.26). Other moderate and positive relationships were observed between FIS with DAI, FUA, RUC, RUH, TEP, and SUS, with values ranging from 0.24 to 0.45, and we found negative correlations between DAI with STR (−0.39) and UDD with FUA (−0.24), RUC (−0.23), and SUS (−0.17).

## DISCUSSION

The results obtained are the first report for this breed for the traits included in this study. A FA1 was used to facilitate the simultaneous analysis for a 18-trait model by using single summary lactation records for SCS, MILK, FAT, and PROT. Here we adopted an approach based on summary lactation information for SCS and milk yield traits. From a practical point of view, lactation summary information is a logical approach to the genetic evaluation of dairy goats, similar to that used by the USDA for the genetic evaluation of dairy goats in the United States [8] and elsewhere [4,15].

Averages for SCS of ND were low, compared to other breeds of dairy goats [4,16,17]. Averages of milk SCS in primiparous goats were 5.1 (±1.4) and 5.3 (±1.2) in the Alpine and Saanen breeds from France, which corresponds to an average SCS of about 1,000,000 cells/mL [4]. Dairy goat averages of SCS were between 4.57 and 5.15×10^3^ cell/mL for Nubian and Toggenburg breeds in the United States [18].

For MILK, FAT, and PROT, ND productions were low compared to other US goat breeds, whose minimum and maximum values ranged from 874 to 1,169 kg for MILK, 37 to 42 kg for FAT and 27 to 35 kg for PROT [11]. The means for MILK were much higher compared to figures for West African Dwarf goats in Nigeria [19], which is to be expected due to the differences in nutritional and management conditions. The averages for fat and protein content in this data were higher compared to other breeds [3,20,21]. The averages and standard deviations obtained for type traits in US goats [22] were not very different than those estimated for some traits in this study. Averages for FIS, FUA, and UDD were similar (84.1, 31.3, 31.2 vs 83.79, 31.57, 31.45, respectively), but different for SUS, RUW, and STA, with values of 19.93, 23.14, 23.3 vs 27.07, 27.01, 27.57, respectively [22]. Coefficients of variation for MILK, FAT, and PROT are within the range of estimates for these traits in other US dairy goat populations [11].

Heritabilities for milk yield traits were lower compared to those estimated in other goat populations [4,23,24], but not unusual [11]. Heritability estimates for ND goats suggest that selection to improve milk yield traits should be successful. Estimated heritabilities in Saanen goats in Mexico were 0.17 for MILK, and 0.19 for FAT, similar to those estimated in ND goats in this study, but were higher for PROT (0.17). Arnal et al [23] estimated average heritabilities for MILK, FAT, and PROT of around 0.24, 0.25, and 0.22, respectively, in Alpine and Saanen breeds from France, using random regression model methodology. Heritabilities for the same traits and order for US goats using a model across breeds, were 0.37, 0.37, and 0.38, respectively [24]. In contrast, in La Mancha and Oberhasli breeds, heritabilities for MILK, FAT, and PROT were very high, with values of 0.48, 0.43, and 0.54, and 0.61, 0.60 and 0.59, respectively [11]. ND herds are generally small, which caused an increased degree of confounding between herd, year and genetic effects. Confounding generally inflates heritability estimates.

Heritability for SCS in ND was slightly higher than those obtained in Alpine and Saanen goats in France (0.20 and 0.24) [4], from 0.09 to 0.22 depending on days in milk for the same breeds [23], and 0.21 for Polish dairy goats [20]. In New Zealand, the heritability estimated for the SCS (SCS = Log_2_ of SCC/1,000) in mixed dairy goats using a random regression model was 0.12 during the first month of lactation and increased to 0.25 at the end of lactation [16]. Scholtens et al [15] obtained a heritability of 0.21 for the average SCS over lactation, calculated as the mean Log_2_ (SCC) from each flock-test, in dairy goats also from New Zealand.

The estimated heritabilities for type traits in the goats of this study were not notably different from those obtained by Luo et al [10] and Castañeda-Bustos et al [22]. Valencia-Posadas et al [25] estimated heritabilities for type traits using a repeatability model in the same breed and the values were different for FIS, DAI, and TED with respect to those estimated in this study. The estimated heritabilities for STR, REL, FUA, RUC, RUA, TEP, and UDD in ND goats had values between 0.13 and 0.34, similar to those obtained by Castañeda-Bustos et al [22] and Luo et al [10]. Heritabilities in ND for STA, TED, and RUW were higher than those estimated by Luo et al [10] with values of 0.52, 0.38, and 0.27, respectively.

Genetic correlations between SCS with milk yield traits similar to those estimated in Alpine and Saanen breeds in France (from −0.13 to 0.12) [23], and f or New Zealand dairy goats (from −0.01 to 0.10) for these same traits [15]. Results for ND were positive but small and not different from zero.

In this study, the values of genetic and phenotypic correlations between milk yield traits were high as is usual in dairy goats [15,21,26,27]. Results of the genetic correlations between SCS and type traits coincide with the estimates by Rupp et al [4] for the Saanen breed, as well as for most genetic correlations in Alpine goats, except for teat length, teat width and teat form with slightly higher values of 0.29, 0.34, and −0.27, respectively.

Because of low genetic correlations between SCS and type traits, options for indirect selection are limited; however, the heritability for SCS was moderate (0.32), therefore direct selection for this trait may be successful.

McLaren et al [28] estimated genetic correlations between MILK and udder traits and leg and feet traits throughout lactation in mixed breed dairy goats using a multi-trait random regression analysis. The genetic correlations estimated between type traits and milk yield across the first lactation demonstrated changes during lactation. Most estimated correlations between milk yield and the udder and teat traits were negative as were those with udder furrow, ranging from −0.42 and 0.18, and correlations between milk yield and UDD followed a similar pattern. Correlations were also observed between milk yield and udder attachment with values ranging from −0.07 to −0.32. Manfredi et al [26] also observed negative correlations ranging from −0.51 to −0.19 when estimating correlations between udder traits and milk yield. Correlations between milk yield with legs and feet start negative in early lactation (approximately −0.20), become positive in mid-lactation (approximately 0.16) and finally fall back to negative values (−0.05) at the end of lactation [28]. In goats from France, Manfredi et al [26] found that most correlations between estimated breeding values (EBVs) for type traits and EBVs for milk yield were low, the exception being the antagonistic association found between EBVs for milk yield and suspensory system traits.

Final score is an important trait because it evaluates the overall type of an animal and therefore is related to linear type traits because it is calculated on the basis of four major categories: general appearance, dairy character, body capacity, and mammary system [3,7]. Final score has been found to be positively related to both productive life and functional productive life in the US multibreed goat population [22] using data that does not include ND. Due to the genetic correlations estimated in this study, a moderate to high antagonistic association was found between FIS and the milk yield traits, but they were not significant.

McLaren et al [28] generally estimated moderate to low genetic correlations between udder characteristics and foot and leg traits but observed particularly high values between udder attachment and UDD (0.77), TEP and teat angle (0.69), TEP and udder attachment (0.57) and teat shape with teat angle (−0.55). In the same study, the authors found the highest correlations between UDD and udder attachment (0.78), teat angle and TEP (0.70), and back legs and back feet (0.64). Valencia-Posadas et al [25] estimated genetic correlations for type traits in seven US goat breeds and for a set of all breeds and found differences between breeds even for the same pair of traits. The highest positive correlations were between STA and RUA (0.76 in ND), DAI and REL (0.54 in Toggenburg), FUA and FIS (0.82 in Oberhasli), RUH and RUA (0.72 in Oberhasli) and between TEP and FIS (0.63 in ND). In the same study the highest negative genetic correlations were between STA and DAI (−0.98 in ND) and between SUS and UDD (−0.63 in Toggenburg). The estimates for STA with RUA, STR with DAI and between TEP with FIS were different than those obtained by Valencia-Posadas et al [25] in the ND breed, possibly due to differences in the pedigree data and models used.

Luo et al [10] found that the genetic correlations between FIS and other type traits in US dairy goats were positive for most of the traits, except for DAI and TED (−0.15 and −0.10), with the highest values found in FUA (0.66), RUC (0.44), RUW (0.36), and STR (0.30). For form traits (STA, STR, and DAI), genetic correlations with other linear traits were generally moderate to small (<0.40), except for STA with RUW (0.63) and RUC (0.44) and STR with DAI (−0.51). The authors also found the largest genetic correlations for SUS with TEP (0.36), TED (0.40), and UDD (−0.34); RUH with RUC (0.38); and TED with TEP (0.34).

The phenotypic correlations estimated by Luo et al [10] among type traits were generally low as well except for FIS with RUC (0.33) and FUA (0.55), between STA and RUW (0.34), SUS and TED (0.34), RUC and RUH (0.30) and STR and DAI (−0.41); all other correlations were <0.29 in absolute terms.

Montaldo and Martínez-Lozano [29] studied phenotypic relationships between udder and milking characteristics, milk yield, and California mastitis test (CMT) results in goats. Milk yield (r = −0.32) and udder perimeter (r = −0.33) were correlated with CMT (p<0.05); udder perimeter had r = 0.81 (p<0.01), and teat perimeter had r = 0.45 (p<0.05) with milk yield.

In another study, Valencia-Posadas et al [30] estimated phenotypic correlations between first lactation milk yield and type traits in Saanen, Alpine and Toggenburg goats and most of them were low. The highest correlations were found between milk yield and UDD (−0.22), STA (0.18), and RUH (0.12) (p<0.05), and the non-linear relationships between STA and STR (p<0.05). In terms of selecting ND goats with a higher production ability, these results indicated that the definition of favorable/unfavorable values for type may need to be redefined.

Considerable multi-trait genetic variation exists in the ND breed for most of the traits considered in this study, which could be expected insofar as it is a breed that has only recently started systematic breeding. However only moderate heritability values were found for MILK and FAT and lower for PROT. It is difficult to determine the reason for these particular heritability values for milk yield traits, but the data structure and the model may have contributed. Strong and positive genetic correlations were found between MILK, FAT, and PROT with STA, SUS, DAI, and RUW, and moderate to highly negative correlations were observed between the same milk yield traits with RUH and RUA, suggesting that our results are different from those found in other dairy goat populations in the US, since some type traits in ND goats are related to milk yield traits. These relationships will need to be confirmed in further studies. The development of selection indices to weight the different traits on a rational basis may help accelerate favorable changes in this breed. The development of genetic programs in this breed could be a factor favoring their increased use in commercial lines of dairy goats to avoid losses of genetic diversity.

In conclusion, substantial genetic variation was observed in many of the studied traits, including SCS, therefore selection can be used for their improvement. Genetic and phenotypic correlations between milk traits were very high as is typical in dairy goats and absolute values of genetic correlations involving SCS with type traits were moderate to low. High and positive genetic correlations were found between DAI, RUW, SUS, and STA with milk yield traits, and high but negative correlations were identified between RUH and FIS with the same milk yield traits. Most of the phenotypic correlations obtained in this study were low.

## Figures and Tables

**Table 1:** Descriptive statistics for studied traits in Nigerian Dwarf goats (n = 1,041)

Trait	Units	Mean	SD	CV%
SCS	Log_2_	2.85	1.29	45.28
MILK	kg	314.05	110.64	35.23
FAT	kg	20.94	7.46	35.62
PROT	kg	14.00	4.87	34.79
FIS	Points	84.10	3.26	3.88
STA	Points	23.31	5.61	24.06
STR	Points	27.99	3.28	11.73
DAI	Points	30.82	3.34	10.82
TED	Points	11.28	6.60	58.52
REL	Points	27.71	3.01	10.88
RUA	Points	27.19	4.63	17.04
RUW	Points	23.14	6.77	29.25
FUA	Points	31.30	3.38	10.79
RUH	Points	30.44	7.04	23.14
RUC	Points	24.46	5.38	21.98
UDD	Points	31.25	6.12	19.60
SUS	Points	19.93	4.37	21.92
TEP	Points	15.91	4.27	26.81

SD, standard deviation; CV%, coefficient of variation; SCS, somatic cell score; MILK, milk yield; FAT, fat yield; PROT, protein yield; FIS, final score; STA, stature; STR, strength; DAI, dairyness; TED, teat diameter; REL, rear legs; RUA, rump angle; RUW, rump width; FUA, fore udder attachment; RUH, rear udder height; RUC, rear udder arch; UDD, udder depth; SUS, medial suspensory ligament; TEP, teat placement.

**Table 2:** Heritability and phenotypic variance estimates for studied traits in Nigerian Dwarf goats

Trait	Heritability	SE	Phenotypic variance
SCS	0.319	0.095	0.92
MILK	0.162	0.078	5,965.10
FAT	0.155	0.084	26.18
PROT	0.102	0.079	11.36
FIS	0.070	0.070	8.19
STA	0.719	0.074	24.88
STR	0.238	0.085	5.80
DAI	0.003	0.071	7.79
TED	0.492	0.099	14.70
REL	0.193	0.086	5.68
RUA	0.139	0.084	12.24
RUW	0.482	0.082	30.78
FUA	0.128	0.077	7.75
RUH	0.069	0.086	13.60
RUC	0.201	0.080	20.40
UDD	0.342	0.087	22.15
SUS	0.029	0.080	13.46
TEP	0.192	0.081	11.64

SE, standard error; SCS, somatic cell score; MILK, milk yield; FAT, fat yield; PROT, protein yield; FIS, final score; STA, stature; STR, strength; DAI, dairyness; TED, teat diameter; REL, rear legs; RUA, rump angle; RUW, rump width; FUA, fore udder attachment; RUH, rear udder height; RUC, rear udder arch; UDD, udder depth; SUS, medial suspensory ligament; TEP, teat placement.

**Table 3:** Genetic (above diagonal) and phenotypic (below diagonal) correlation estimates for somatic cell score, milk yield traits and type traits in Nigerian Dwarf goat

Trait	Trait																	

	SCS	MILK	FAT	PROT	FIS	STA	STR	DAI	TED	REL	RUA	RUW	FUA	RUH	RUC	UDD	SUS	TEP
SCS	-	0.25	0.18	0.23	−0.13	0.18	0.09	0.17	0.06	0.00	−0.06	0.19	−0.04	−0.16	−0.02	0.06	0.22	0.01
MILK	−0.06	-	**0.72**	**0.91**	−0.52	**0.72**	0.37	0.70	0.24	0.02	−0.24	**0.76**	−0.17	−0.64	−0.06	0.24	0.88	0.02
FAT	−**0.10**	**0.85**	-	**0.66**	−0.38	**0.52**	0.27	0.51	0.17	0.01	−0.17	**0.55**	−0.12	−0.47	−0.05	0.18	0.64	0.01
PROT	−**0.08**	**0.93**	**0.84**	-	−0.48	**0.65**	0.34	0.64	0.22	0.02	−0.22	**0.70**	−0.15	−0.59	−0.06	0.22	0.81	0.02
FIS	0.03	**0.25**	**0.21**	**0.24**	-	−0.37	−0.19	−0.36	−0.12	−0.01	0.13	−0.40	0.09	0.33	0.03	−0.13	−0.46	−0.01
STA	**0.07**	**0.33**	**0.27**	**0.30**	**0.13**	-	**0.27**	0.50	0.17	0.01	−0.17	**0.55**	−0.12	−0.46	−0.05	0.18	0.63	0.01
STR	**0.06**	**0.10**	**0.10**	**0.09**	**0.10**	**0.15**	-	0.26	0.09	0.01	−0.09	0.29	−0.06	−0.24	−0.03	0.08	0.33	0.01
DAI	−0.03	**0.19**	**0.14**	**0.17**	**0.34**	0.03	−**0.39**	-	0.17	0.01	−0.17	0.53	−0.12	−0.45	−0.05	0.17	0.62	0.01
TED	−0.03	**0.10**	**0.11**	**0.09**	0.04	**0.13**	**0.07**	−0.02	-	0.00	−0.06	0.18	−0.04	−0.15	−0.01	0.06	0.21	0.01
REL	0.01	0.01	0.00	0.00	**0.14**	−0.01	−**0.06**	**0.15**	−0.05	-	0.00	0.01	0.00	−0.01	0.00	0.00	0.02	0.00
RUA	0.04	−0.02	−0.05	−0.03	**0.27**	0.04	0.01	**0.24**	0.03	0.03	-	−0.18	0.04	0.15	0.02	−0.06	−0.21	−0.01
RUW	0.05	**0.18**	**0.16**	**0.15**	**0.14**	**0.41**	**0.30**	−**0.07**	**0.15**	−0.01	**0.11**	-	−0.13	−0.49	−0.05	0.18	0.67	0.01
FUA	−0.03	**0.31**	**0.28**	**0.31**	**0.45**	0.05	**0.15**	**0.13**	−0.02	0.03	0.01	0.04	-	0.11	0.01	−0.04	−0.15	−0.01
RUH	0.01	0.06	0.02	0.04	**0.34**	−0.03	−**0.08**	**0.16**	0.06	−0.01	**0.06**	−**0.07**	**0.19**	-	0.04	−0.16	−0.57	−0.01
RUC	0.03	**0.25**	**0.19**	**0.25**	**0.42**	0.06	**0.08**	**0.15**	0.01	0.02	**0.09**	**0.07**	**0.30**	**0.19**	-	−0.02	−0.06	0.00
UDD	0.03	−**0.24**	−**0.25**	−**0.27**	−**0.10**	0.05	−**0.14**	−0.01	−0.05	0.02	0.03	0.01	−**0.24**	0.04	−**0.23**	-	0.21	0.01
SUS	0.00	**0.15**	**0.13**	**0.12**	**0.24**	**0.06**	**0.13**	0.01	**0.25**	−0.02	**0.06**	**0.14**	**0.07**	**0.08**	**0.11**	−**0.17**	-	0.02
TEP	0.03	0.00	0.00	0.02	**0.35**	0.04	0.04	0.02	**0.12**	**0.10**	**0.09**	0.01	**0.10**	**0.11**	**0.24**	−0.01	**0.32**	-

SCS, somatic cell score; MILK, milk yield; FAT, fat yield; PROT, protein yield; FIS, final score; STA, stature; STR, strength; DAI, dairyness; TED, teat diameter; REL, rear legs; RUA, rump angle; RUW, rump width; FUA, fore udder attachment; RUH, rear udder height; RUC, rear udder arch; UDD, udder depth; SUS, medial suspensory ligament; TEP, teat placement.

Estimates in boldface, p<0.05.
